# Synthesis of New Water Soluble β-Cyclodextrin@Curcumin Conjugates and In Vitro Safety Evaluation in Primary Cultures of Rat Cortical Neurons

**DOI:** 10.3390/ijms22063255

**Published:** 2021-03-23

**Authors:** Amina Ben Mihoub, Samir Acherar, Céline Frochot, Catherine Malaplate, Frances T. Yen, Elmira Arab-Tehrany

**Affiliations:** 1 LIBio Laboratory, Université de Lorraine, F-54000 Nancy, France; 2 LCPM, CNRS, Université de Lorraine, F-54000 Nancy, France; 3 LRGP, CNRS, Université de Lorraine, F-54000 Nancy, France; celine.frochot@univ-lorraine.fr; 4 URAFPA, INRAE, Université de Lorraine, F-54000 Nancy, France; catherine.malaplate-armand@univ-lorraine.fr (C.M.); frances.yen-potin@univ-lorraine.fr (F.T.Y.)

**Keywords:** curcumin, β-cyclodextrin, click chemistry, nanoconjugates, neurodegenerative prevention

## Abstract

Self-aggregation of Curcumin (Cur) in aqueous biological environment decreases its bioavailability and in vivo therapeutic efficacy, which hampers its clinical use as candidate for reducing risk of neurodegenerative diseases. Here, we focused on the design of new Cur- β-Cyclodextrin nanoconjugates to improve the solubility and reduce cell toxicity of Cur. In this study, we described the synthesis, structural characterization, photophysical properties and neuron cell toxicity of two new water soluble β-CD/Cur nanoconjugates as new strategy for reducing risks of neurodegenerative diseases. Cur was coupled to one or two β-CD molecules via triazole rings using CuAAC click chemistry strategy to yield β-CD@Cur and (β-CD)_2_@Cur nanoconjugates, respectively. The synthesized nanoconjugates were found to be able to self-assemble in aqueous condition and form nano-aggregates of an average diameter size of around 35 and 120 nm for β-CD@Cur and (β-CD)_2_@Cur, respectively. The photophysical properties, water solubility and cell toxicity on rat embryonic cortical neurons of the designed nanoconjugates were investigated and compared to that of Cur alone. The findings revealed that both new nanoconjugates displayed better water solubility and in vitro biocompatibility than Cur alone, thus making it possible to envisage their use as future nano-systems for the prevention or risk reduction of neurodegenerative diseases.

## 1. Introduction

Neurodegenerative diseases, such as Parkinson’s and Alzheimer’s diseases, as well as spinocerebellar ataxias, are common causes of cognitive impairment and morbidity in older adults [1,2]. The heterogeneous clinical presentation and physiopathology of the neural disorders that characterize these diseases can also be associated with inflammation, oxidative damage and the protein aggregate formation [3,4]. Epidemiological studies point towards natural antioxidant agents as promising therapeutic agents to prevent or delay the occurrence of neurodegenerative diseases [5].

Curcumin (Cur) is the major polyphenolic compound of Curcuma longa, an herbaceous plant endemic to South Asia [6]. There have been numerous studies on the bioactivity and health benefits of Cur, including its antioxidant, anti-inflammatory, antitumor and neuroprotective effects [7]. In recent years, this drug has been considered as a potential treatment for multiple neurodegenerative disorders [8]. Moreover, multiple studies in rodents and humans have shown that Cur crosses the blood–brain barrier (BBB) [5]. However, despite its many biological activities, Cur is not often used in clinical applications because of two main intrinsic properties: Low bioavailability due to its limited solubility in water (11 ng/mL) and low permeability (LogP 3.28) [9]. 

Recently, Cur-based nano-systems, including liposomes, polymeric nanoparticles, micelles, conjugates, solid dispersion, emulsion, peptide carriers and cyclodextrins (CDs) have been proposed as suitable and promising systems for enhancing the overall bioavailability, biodistribution and controlled release of Cur, along with reducing Cur toxic effects, and increased transport across the BBB [4,7,9,10,11,12]. Because of their unique physicochemical and structural properties, CDs were considered as one of the most promising biocompatible nanovectors for Alzheimer’s disease (AD) [13,14]. CDs (α-, β-, and δ-CD) are a family of natural glucopyranose-based cyclic oligosaccharides, with hydrophobic internal and hydrophilic external surfaces by the exposure of hydroxyl units [10]. CDs and their derivatives proved to form complexes with Cur [15,16,17,18,19,20,21], improving its water solubility by around 100 times, its bioactivity [15,16,17,18] and its anti-AD therapeutic effect [19,20,21]. To our knowledge, the improvement of Cur water solubility and anti-AD therapeutic efficacy with CDs has been investigated, but only in cases of complex formation through non-covalent binding.

**Figure 1:**
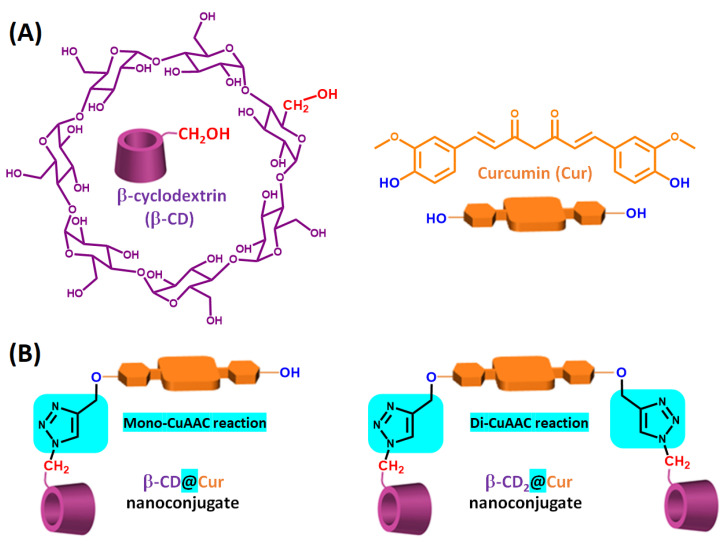
(**A**) Chemical structures of β-CD and Cur, and (**B**) schematic illustration of β-CD@Cur and (β-CD)_2_@Cur nanoconjugates obtained through mono- and di-CuAAC reaction.

In this current study, we explored two new nanoconjugates based on the covalent binding of Cur to either one or two β-CD molecules identified as β-CD@Cur and (β-CD)_2_@Cur, respectively (Figure 1). The two nanoconjugates were synthesized using Copper-Catalyzed Azide-Alkyne Cycloaddition (CuAAC) click chemistry. They were characterized by DLS, NMR (^1^H, ^13^C, HMBC and HSQC), HRMS and HPLC techniques. The effect of the covalent binding on Cur disaggregation in aqueous conditions was investigated by spectroscopic measurements. Finally, cytotoxic studies were performed on primary cultures of rat embryonic cortical neurons to evaluate the behavior of the nanoconjugates in a biological environment.

## 2. Results and Discussion

### 2.1. Synthesis of β-CD@Cur ***4*** and (β-CD)_2_@Cur ***5*** Nanoconjugates

**Scheme 1:**
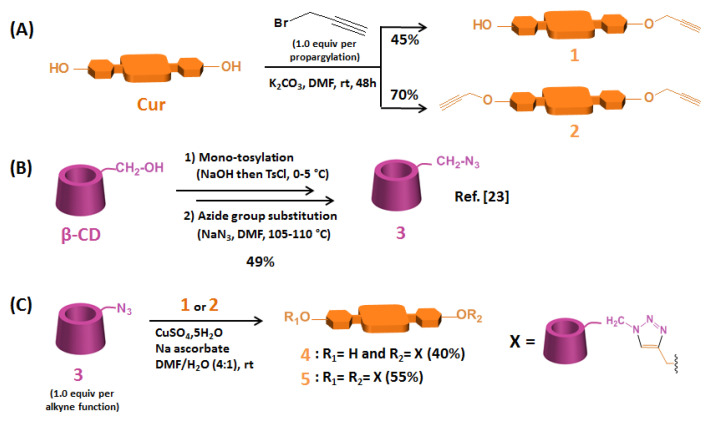
Synthesis of (**A**) Cur mono-alkyne **1** and Cur di-alkyne **2**, (**B**) mono-6-azido-β-CD **3**, and (**C**) β-CD@Cur **4** and (β-CD)_2_@Cur **5** nanoconjugates.

For the synthesis of the nanoconjugates β-CD@Cur **4** and (β-CD)_2_@Cur **5**, Cur was combined with β-CD via a triazole covalent linkage using a CuAAC click chemistry strategy. To achieve this goal, Cur mono-alkyne **1**, Cur di-alkyne **2** and mono-6-azido-β-CD **3** were first synthesized as previously described [22,23] (Scheme 1).

The obtained yields of Cur mono-alkyne **1** and Cur di-alkyne **2** were relatively good (45% and 70%, respectively). These compounds were synthesized separately by mono- and di-etherification of Cur with propargyl bromide (1.0 equiv. per propargylation) in alkaline conditions using a previous protocol described by Raja et al. [22] with slight modifications (Scheme 1A). Compounds **1** and **2** were characterized by HPLC, ^1^H NMR and HRMS (see supplementary data).

Mono-6-azido-β-CD **3** was prepared in two steps from β-CD according to the protocol previously described by our team [23] with a 49% overall yield (Scheme 1B). These two steps consisted of mono-tosylation of β-CD on the primary OH-6 function, followed by its nucleophilic substitution by an azide group.

β-CD@Cur **4** and (β-CD)_2_@Cur **5** resulting from single and double “click” conjugations between alkyne-modified Cur (**1** or **2**) and azide-functionalized β-CD **3** were obtained as an orange powder and as a yellowish powder with 40% and 55% yields, respectively (Scheme 1C).

**Figure 2:**
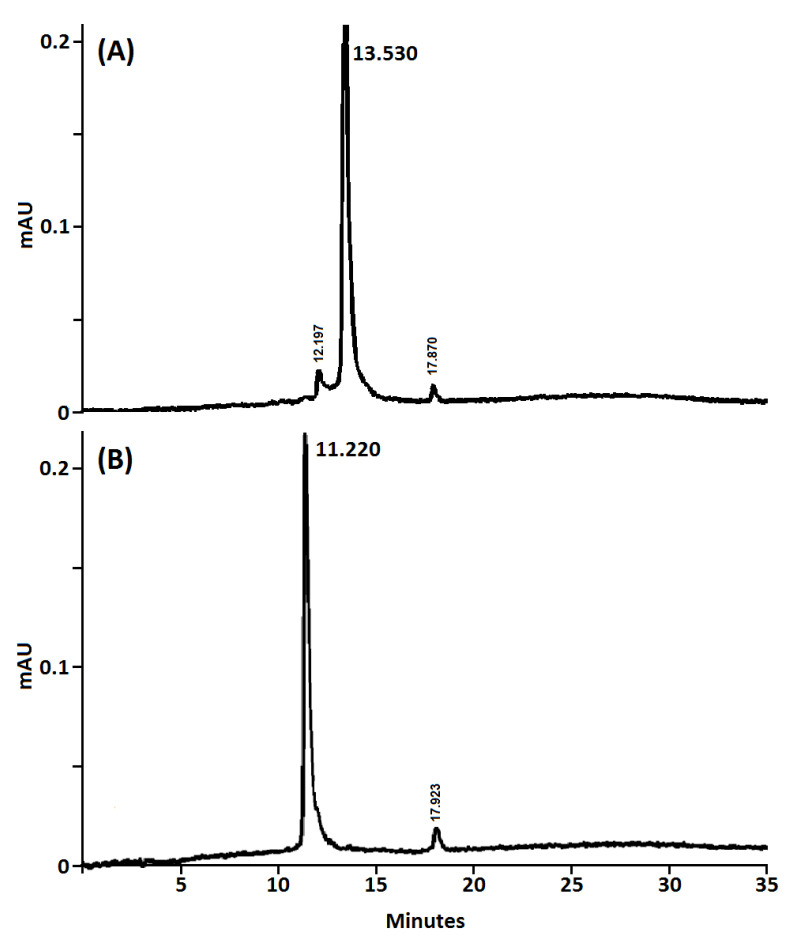
Analytical HPLC chromatograms (UV detection at 420 nm) of (**A**) β-CD@Cur **4** and (**B**) (β-CD)_2_@Cur **5** nanoconjugates (C18 column, ACN/H_2_O containing 0.1% TFA, *v/v* gradients from 10/90 to 100/0 in 25 min, then 100/0 for 10 min).

The purity of the obtained nanoconjugates was determined by analytical HPLC and NMR analysis. The HPLC purity of both nanoconjugates was determined using reverse phase and based on the UV-visible detection (254 and 420 nm). As shown in Figure 2, both β-CD@Cur **4** and (β-CD)_2_@Cur **5** nanoconjugates showed an excellent purity (94.2% and 95.3% for **4** and **5**, respectively).

The purity and the successful synthesis of both nanoconjugates were characterized with 1D and 2D NMR (^1^H, ^13^C, HSQC and HMBC) and HRMS analyses. All 1D and 2D NMR spectra of nanoconjugates **4** and **5** are included in supplemental data (Figures S1–S8). In the ^1^H NMR spectra of β-CD@Cur **4** and (β-CD)_2_@Cur **5** (Figures S1 and S5), the characterization was based on the complete clearance of the propargylic CH signal at 3.59 ppm and the appearance of the characteristic signal of the triazole ring at around 8.20 ppm. The obtention of the mono-CuAAC reaction was also evidenced in NMR spectra of β-CD@Cur **4** by the presence of (i) one hydroxyl group at 9.67 ppm in the ^1^H NMR spectrum (i.e., one phenol group in Cur unit, Figure S1) and (ii) duplicated signals in the ^1^H and ^13^C NMR spectra (i.e., H**_3_**/H**_3′_**, H**_4_**/H**_4′_**, H**_6_**/H**_6′_**, H**_9_**/H**_9′_** and H**_10_**/H**_10′_** in the ^1^H NMR spectrum, Figure S1, and C**_2_**-C**_10_**/C**_2′_**-C**_10′_** in the ^13^C NMR spectrum, Figure S2) due to the presence of dissymmetry in β-CD@Cur **4**. Compared to β-CD@Cur **4**, in the ^1^H and ^13^C NMR spectra of (β-CD)_2_@Cur **5** (Figures S5 and S6), the di-CuAAC reaction was evidenced by the disappearance of (i) the ^1^H NMR signal of the hydroxyl group (i.e., no phenol group in Cur unit, Figure S5) and (ii) the duplication of signals in ^1^H and ^13^C NMR spectra (Figures S5 and S6). Finally, the ^1^H and ^13^C NMR signals of **4** and **5** were fully assigned using 2D NMR experiments (Figures S3, S4, S6 and S8).

### 2.2. Size and Morphological

**Figure 3:**
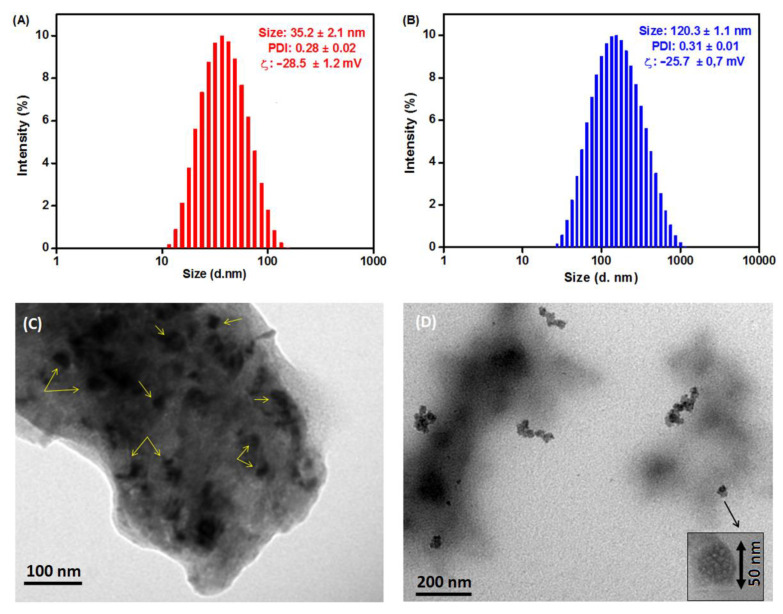
DLS profiles in water of (**A**) β-CD@Cur **4** and (**B**) (β-CD)_2_@Cur **5**. TEM images of (**C**) β-CD@Cur **4** and (**D**) (β-CD)_2_@Cur **5**.

To investigate the size and morphology of the developed β-CD@Cur **4** and (β-CD)_2_@Cur **5** nanoconjugates, DLS and TEM analyses were performed. As shown in Figure 3, the synthesized supramolecular nanoconjugates displayed a tendency to self-assemble in water due to the strong interaction between Cur and β-CD (O-H bonds, Host-Guest, etc.). The self-assembled β-CD@Cur **4** had an average hydrodynamic diameter of 35.2 ± 2.1 nm in the DLS profile and zeta potential of −28.5 ± 1.2 mV (Figure 3A). The self-assembled (β-CD)_2_@Cur **5** showed a larger mean diameter of 120.3 ± 1.1 nm and zeta potential of −25.7 ± 0.7 mV (Figure 3B). This increase in the mean diameter may be accounted by the presence of the double amount of β-CD in nanoconjugate **5** compared to **4**.

As depicted in TEM images (Figure 3C,D), β-CD@Cur **4** and (β-CD)_2_@Cur **5** showed spherical morphology with an average size of around 25 nm for β-CD@Cur **4** (Figure 3C) and 50 nm spheres aggregated into chains of diameter size around 100 nm (β-CD)_2_@Cur **5** (Figure 3D). The size differences obtained by both DLS and TEM are possible, since DLS and TEM are two different techniques. Indeed, DLS provides the hydrodynamic diameter of swollen vesicles in solution [24]. TEM allows to obtain two-dimensional image of the dried form [25]. This size difference could be also linked to the fact that we obtain relatively high PDI values of around 0.3 for both nanoconjugates resulting in a wide distribution of sizes and different size visualization in TEM images.

### 2.3. β-CD Effect on the Cur Disaggregation in Aqueous Medium

Cur is a natural hydrophobic photosensitizer having a low water solubility (i.e., 11 ng/mL [9]). Generally, hydrophobic photosensitizers have the tendency to self-aggregate in aqueous media due to their conjugated π–π electronic structure resulting in the obtention of broad UV–vis absorption peaks with low intensity and quenching fluorescence emission. In the case of Cur, experimental study on the Cur aggregation revealed that its aggregation in water is due to π-stacking of phenyl rings and H-bonds with side groups [26], leading to a red Stockes shift by comparing UV–vis absorption and fluorescence emission spectra [27]. It is worth noting that β-CD is a natural cyclic oligosaccharide consisting of α-1,4-glucopyranose units. In contrast with curcumin, β-CD does not have a conjugated π–π electronic structure, with the result that β-CD does not absorb and fluoresce in the UV–vis. β-CD is biocompatible and biodegradable oligosaccharide known to improve the solubility, stability and bioavailability of drugs.

**Figure 4:**
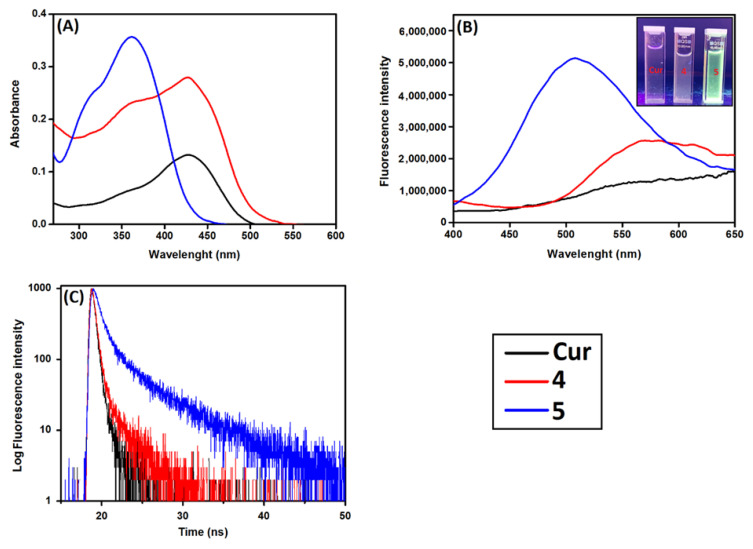
Photophysical properties of Cur, β-CD@Cur **4** and (β-CD)_2_@Cur **5** in D_2_O at room temperature. (**A**) UV–vis absorption spectra at a single concentration of Cur (c = 12 μM), and (**B**) fluorescence emission and (**C**) fluorescence lifetime spectra at λ_exc_ = 360 nm (A_360 nm_ = 0.2).

The effect of β-CD on the disaggregation of Cur in aqueous medium was investigated by comparing the photophysical properties of nanoconjugates **4** and **5** to those of the free Cur in water. The UV–vis absorption, fluorescence emission and fluorescence lifetime spectra in water of the studied molecules are shown in Figure 4. As shown in Figure 4A, in aqueous conditions (neutral water), Cur exhibited a broad absorption band at around 427 nm with a shoulder at ∼350 nm, which arise assigned to the π−π* transitions associated with the conjugated Cur and the feruloyl unit, respectively [28,29].

By covalent binding of Cur to β-CD molecules, two important changes in the UV–vis absorption spectra of Cur were observed. First, a hyperchromic effect was observed in the UV–vis spectra of both nanoconjugates in D_2_O, where the absorption intensity of Cur in water increased significantly depending on the number of β-CD molecules coupled to Cur (Figure 4A), indicating an increase in the solubilization of Cur inside the nanoconjugates [28,29,30]. Second, the broad absorption band at 427 nm became narrower in the case of both nanoconjugates and a significant blue shift to 365 nm occurred in the case of the nanoconjugate **5** (Figure 4A). Such changes might be due to the Cur π-electrons interaction with the hybridized orbitals of the surface oxygen atoms of β-CD, leading to destabilization of the π*-orbitals, and a stabilization of the π-orbitals causing a blue shift of the absorption band of the coupled Cur [29]. 

The effect of β-CD on the fluorescence intensity and lifetime of Cur in D_2_O was also investigated. As shown in Figure 4B, Cur had a tendency to quench in aqueous media leading to weak fluorescence intensity with a broad emission spectrum centered at 550 nm and fluorescence lifetime of 1.6 ns (Figure 4C, Table 1). However, by coupling Cur to β-CD, a significant enhancement of fluorescence intensity occurred depending on the number of β-CD coupled to Cur with an important blue-shift in the emission maxima in the case of nanoconjugate 5 (Figure 4B). This effect could be visually observed under a UV light of 365 nm (top right of Figure 4B). Moreover, fluorescence quantum yield and fluorescence lifetime increased by increasing covalent binding of Cur to β-CD (Figure 4C, Table 1). This might be due to a decrease of the aggregation and the presence of the nanoconjugates monomers in water. Such results demonstrated that: (i) Covalent binding of Cur to β-CD decreased its self-aggregation in aqueous conditions and (ii) the disaggregation phenomenon was highly dependent on the number of β-CD units linked to Cur (i.e., the more there are, the better).

### 2.4. Treatment of Primary Cultured Neurons with Nanoconjugates ***4*** and ***5***

**Figure 5:**
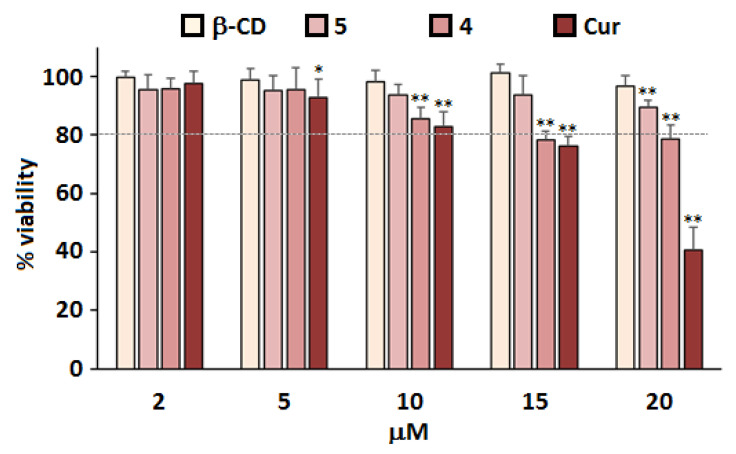
Cytotoxicity of β-CD@Cur **4** and (β-CD)_2_@Cur **5** nanoconjugates. Primary cultures of rat embryo cortical neurons were isolated and prepared on Day 0 (D0) in vitro, followed by treatment on D3 with the indicated concentrations of Cur. MTT assay was performed after 24 h to assess cell viability. Results are shown as % of viability of control cells treated with the same volume of DMSO alone; means and SD are shown for two separate cultures each with quadruplicate determinations. Statistical significance is shown as compared to values obtained from control cells (* *p* < 0.05, ** *p* < 0.01).

The cytotoxic effect of the synthesized nanoconjugates **4** and **5** on primary cultures of rat embryo cortical neurons was investigated (the day of cell isolation and preparation of culture was Day 0 (D0)). To assess cytotoxic effects of β-CD@Cur **4** and (β-CD)_2_@Cur **5** nanoconjugates, primary cultures of neurons were prepared followed by 24 h incubation starting on D3 in the absence or presence of the nanoconjugates at Cur concentrations of 2, 5, 10, 15 and 20 µM. Cell viability (Figure 5) was determined by the MTT assay on D4 as previously described [31,32].

In Figure 5, Cur showed a drug concentration-dependent cytotoxicity to rat cortical neurons cells. In fact, a significant decrease in cell viability was observed at Cur levels starting from 10 µM; only 48% of cells remained viable after treatments with 20 µM Cur. Such results were probably due to the fact that Cur molecules were not well dispersed throughout the cell medium due to its lack of water solubility [24]. However, after click-covalent binding of Cur with β-CD, no significant cytotoxicity to rat cortical neurons cells at corresponding Cur concentrations was observed. The degree of improved cell viability was also a function of β-CD molecules number coupled with Cur viability (β-CD@Cur **4** < (β-CD)_2_@Cur **5**). These results were consistent with the photophysical properties of the studied molecules (Section 3.3), therefore suggesting that covalent binding of Cur to β-CD could promote its water solubility and reduce Cur toxicity in primary cultures of rat cortical neurons.

## 3. Materials and Methods

### 3.1. Chemicals

All chemicals were used without further purifications. β-cyclodextrin (β-CD) and curcumin (Cur) were purchased from Sigma-Aldrich (Saint-Quentin Fallavier, France). All reactions involving Cur compounds were protected from the light by using aluminum foil around the reactors and other glassware. Deionized (DI) water was processed in a Milli-Q water purification system.

### 3.2. Characterization

The monitoring of the mono- and bi-propargylation of Cur was carried out using Macharey -Nagel ALUGRAM^®^ SIL > G/UV254 thin-layer chromatography (TLC) plates and spots were visualized under ultraviolet (UV) light (256 nm and 355 nm). Cur-based products purifications were performed on Agilent 1100 high-performance liquid chromatography (HPLC) (Agilent, Santa Clara, CA, USA) using an Agilent Pursuit 5 C18 reverse phase column (5 µm, 150 × 4.6 mm) eluted with acetonitrile/water (ACN/H_2_O, *v/v*) gradients from 10/90 to 100/0 in 25 min, then 100/0 for 10 min containing 0.1% trifluoroacetic acid (TFA). A flow rate of 1.0 mL/min for analytical HPLC and of 12 mL/min for preparative HPLC were used with UV detection at 254 nm and 420 nm. A Brucker Advance 300 MHz spectrophotometer was used to perform 1D and 2D NMR spectra (i.e., ^1^H, ^13^C, HSQC and HMBC). Deuterated DMSO with residual peaks at δ = 2.5 and 39.5 ppm for ^1^H and ^13^C NMR, respectively, was chosen for NMR experiments at room temperature (T = 298 K). δ and *J* represented chemical shifts and coupling constants in parts per million (ppm) and hertz (Hz), respectively. Singlet (s), doublet (d), triplet (t), multiplet (m), broad (br) or their combination defined the multiplicity. High-resolution mass spectroscopy with electrospray ionization method (ESI-HRMS) was performed using a Brucker MicrOTOF-Q HR spectrometer. Ultraviolet-Visible (UV–vis) absorption and fluorescence spectra were recorded on a Shimadzu UV-3600 double-beam UV–vis spectrophotometer and a Fluorolog-3 spectrofluorometer FL3-222, respectively.

### 3.3. Synthesis of β-CD@Cur ***4*** and (β-CD)_2_@Cur ***5*** Nanoconjugates

#### 3.3.1. Cur Mono-Alkyne **1** and Cur Di-Alkyne **2**

Compounds **1** and **2** were synthesized separately as described in the literature [22] with slight modifications by reacting Cur (200 mg, 0.543 mmol) in DMF (3 mL) with K_2_CO_3_ and propargyl bromide (1.0 equiv. per propargylation) at room temperature under N_2_ for 48 h. To avoid degradation of Cur, the reactions were performed in the dark. The progress of the reactions was monitored by TLC with EtOAc/hexane (50/50). After completion of the reactions, the solvent was removed under reduced pressure. The crude products were purified by preparative HPLC and the collected pure fractions were lyophilized over 24 h to yield **1** (45%) and **2** (70%) as orange powders. Compounds **1** and **2** were characterized by HPLC, ^1^H NMR and HRMS (See supplementary data).

#### 3.3.2. β-CD@Cur **4** and (β-CD)_2_@Cur **5** Nanoconjugates

Mono-6-azido-β-CD **3** was synthesized from β-CD in a two steps-process. The overall yield was 49%. The protocol has been described recently by our team [23].

To a solution of Cur mono-alkyne **1** or Cur di-alkyne **2** (0.08 mmol, 1.0 equiv.) and **3** (1.0 equiv. per alkyne function) in H_2_O/DMF (1/4, *v/v*, 5 mL) was added successively CuSO_4_·5H_2_O (0.1 equiv. per alkyne function) and Na ascorbate (0.5 equiv. per alkyne function). The reaction mixtures were maintained at room temperature under vigorous stirring in the dark for 24 h. The solvent was evaporated under reduced pressure. Preparative HPLC was used to purify the crude product and the collected pure fractions were lyophilized over 24 h to afford β-CD@Cur **4** (40%) and (β-CD)_2_@Cur **5** (55%) as an orange powder and a yellowish powder, respectively. 

β-CD@Cur **4**: ^1^H NMR (300 MHz, DMSO-*d*_6_): δ 2.85–4.45 (m, 54H, H2, H3, H4, H5, H6, OH6, H7-OMe and H7′-OMe), 4.48–5.26 (m, 13H, H1, OH2, OH3, H1 and H11), 5.26–6.08 (3, 12H, OH2 and OH3), 6.75 (d, *J* = 8.4 Hz, 1H, H9′), 6.76 (d, *J* = 15.9 Hz, 1H, H3′), 6.81 (d, *J* = 15.6 Hz, 1H, H3), 6.83 (d, *J* = 8.4 Hz, 1H, H9), 7.14 (d, *J* = 8.4 Hz, 1H, H10), 7.21 (br s, 1H, H6′), 7.29 (d, *J* = 8.4 Hz, 1H, H10′), 7.32 (br s, 1H, H6), 7.56 (d, *J* = 15.9 Hz, 1H, H4′), 7.58 (d, *J* = 15.6 Hz, 1H, H4), 8.19 (s, 1H, H13), 9.68 (br s, 1H, H8′-OH). ^13^C NMR (75 MHz, DMSO-*d*_6_): δ 50.4 (C11), 55.6 (C7-OMe and C7′-OMe), 59.1 (C6), 59.9 (C6), 61.5 (C1), 70.0 (C2, C3 and C5), 72.1 (C2, C3 and C5), 72.4 (C2, C3 and C5), 73.0 (C2, C3 and C5), 81.5 (C4), 82.1 (C4), 83.4 (C4), 100.9 (C1), 101.9 (C1), 111.4 (C6), 113.2 (C6′), 115.7 (C9 and C9′), 121.1 (C3′), 12.3 (C3), 122.7 (C10′), 123.2 (C10), 125.8 (C13), 126.3 (C5′), 128.0 (C5), 140.1 (C4′), 141.0 (C4), 142.2 (C12), 148.0 (C8′), 149.2 (C7), 149.4 (C7′), 149.7 (C8), 182.7 (C2 and C2′). HRMS (ESI) for C_66_H_91_N_3_O_40_: [M + H]^+^ calculated 1566.5257, found 1566.5531, [M + Na]^+^ calculated 1588.5076, found 1588.5293 and [M + 2H]^2+^ calculated 783.7667, found 783.7844.

(β-CD)_2_@Cur **5**: ^1^H NMR (300 MHz, DMSO-*d*_6_): δ 3.04–3.75 (m, 93H, H2, H3, H4, H5, H6 and OH6), 3.78 (s, 6H, H7-OMe), 3.95–4.07 (m, 3H, OH6), 4.47–5.62 (m, 48H, H1, OH2, OH3, H1 and H11), 7.00 (d, *J* = 15.3 Hz, 2H, H3), 7.20 (d, *J* = 8.4 Hz, 2H, H9), 7.31 (d, *J* = 8.4 Hz, 2H, H10), 7.37 (s, 2H, H6), 7.61 (d, *J* = 15.3 Hz, 2H, H4), 8.17 (s, 2H, H13). ^13^C NMR (75 MHz, DMSO-*d*_6_): δ 50.4 (C11), 55.7 (C7-OMe), 59.0 (C6), 59.9 (C6), 61.5 (C1), 70.0 (C2, C3 and C5), 71.7 (C2, C3 and C5), 72.1 (C2, C3 and C5), 72.4 (C2, C3 and C5), 73.0 (C2, C3 and C5), 81.1 (C4), 81.5 (C4), 82.1 (C4), 83.4 (C4), 101.3 (C1), 102.0 (C1), 111.3 (C6), 112.9 (C9), 118.8 (C3), 124.1 (C10), 125.8 (C13), 127.0 (C5), 142.0 (C12), 144.7 (C4), 149.1 (C8), 150.5 (C7), 193.1 (C2). HRMS (ESI) for C_111_H_162_N_6_O_74_: [M + 2H]^2+^ calculated 1382.4627, found 1382.5022, [M + 2Na]^2+^ calculated 1404.4446, found 1404.4793 and [M + 2K]^2+^ calculated 1420.4186, found 1420.4437.

### 3.4. Nanoconjugates Size and ζ-Potential Measurements

The size, polydispersity index (PDI) and ζ-potential of the synthesized nanoconjugates were measured following their dilution with ultrapure water (1:200) using DLS (Zetasizer Nano ZS, Malvern, UK). Nanoconjugates were examined by standard capillary electrophoresis cell. The size and ζ-potential were measured at room temperature (25 °C) using a 0.01 absorbance under a scattering angle and a refractive index of 173° and 1.59, respectively.

### 3.5. Nanoconjugates Morphological Study

The morphology of the synthesized nanoconjugates in water was monitored via TEM using a negative staining method. In brief, the nanoconjugates were first diluted in distilled water. A 2% ammonium molybdate solution was added with a ratio of 1:1. The mixtures were reserved at room temperature for 3 min. Then, one drop of the nanoconjugates suspension was deposited and dried on a grid type EMS CF200-Cu Formvar carbon-coated copper grid (200 mesh, 3 mm diameter HF 36). The nanoconjugates morphology was studied with a Philips CM20 TEM equipped with an Olympus TEM CCD camera at 200 kV.

### 3.6. β-CD Effect on Cur Disaggregation in Aqueous Medium

The influence of the presence of β-CD on the solubility of Cur in β-CD@Cur **4** and (β-CD)_2_@Cur **5** nanoconjugates in water at room temperature was studied by comparing their UV–vis absorption, fluorescence emission and fluorescence lifetime spectra with those of free Cur in the same conditions. Briefly, each product was dissolved in D_2_O. Fluorescence (Φ_f_) quantum yield was determined using Quinine (Quin) solution in acetonitrile as fluorescence standard (Φ_f_ = 0.55) [33]. 

### 3.7. Cell Cytotoxicity Studies

#### 3.7.1. Primary Culture of Cortical Neurons

Cell culture studies were carried out at the Bioavailability–Bioactivity (Bio-DA, Lorraine, France) platform. Primary cultures of cortical neurons were prepared from rat fetuses (day 16–17 of gestation) as previously reported [31,32]. Cells were plated at 10 × 10^4^ cells/cm^2^ onto plates and cultured in neuronal culture medium M2 [DMEM-F12 (Invitrogen, Illkirch, France) medium containing 0.5 µM insulin, 60 µM putrescine, 30 nM sodium selenite, 100 µM transferrin, 10 nM progesterone and 0.1% (*w/v*) ovalbumin (Sigma-Aldrich (Saint-Quentin Fallavier, France)]. Neurons were incubated at 35 °C in 6% CO_2_ with day (D) 0 being the day of the preparation of the cells. β-CD, Cur, β-CD@Cur **4** and (β-CD)_2_@Cur **5** were first dissolved in DMSO (Final volume of DMSO ≤ 0.25%), and the solutions were diluted to the desired concentrations in M2 culture medium. On Day 3, the neuronal culture medium was treated with each compound separately at a final Cur concentration of 2–20 µM and incubated for 24 h at 35 °C in 6% CO_2_. Control cells were treated with the same concentrations of DMSO alone that are not toxic for cells.

#### 3.7.2. MTT Test

The neuronal viability of the different molecules was assessed using the 3-(4,5 dimethylthiazol-2-yl)-2,5-diphenyltetrazolium bromide (MTT, Sigma-Aldrich (Saint-Quentin Fallavier, France)) reduction activity assay. MTT solution (4 mg/mL, 37 °C) was added to each well and the plates were then incubated for 1 h at 35 °C in 6% CO_2_. Media was removed and replaced with DMSO followed by incubation while shaking at room temperature for 10 min. The absorbance of formazan was measured at wavelength of 570 nm [(Fluostar Galaxy, Biolise software) (BMG Lab technologies, Offenberg, Germany)]. Cell viability was expressed as percentage of living cells compared to values obtained from control untreated cells.

#### 3.7.3. Statistical Analysis

Experiments were performed in triplicate, and results are shown as the mean ± SD, unless otherwise indicated. A variance analysis was done by ANOVA supplemented with a Scheffe’s post hoc test and a *p*-value greater than 0.05 was regarded as significant.

## 4. Conclusions

Two novel nanoconjugates denoted β-CD@Cur and (β-CD)_2_@Cur self-assembled in water to form nanoparticles with an average diameter size of around 35 and 120 nm, respectively. These nanoconjugates were successfully synthesized using CuAAC click chemistry strategy via a triazole covalent link between Cur and β-CD molecules. The chemical structure and purity of the synthesized nanoconjugates was checked by NMR spectroscopy (^1^H, ^13^C, HSQC and HMBC), ESI-HRMS and HPLC analyses. Furthermore, the β-CD effect on the disaggregation of Cur in aqueous medium and cell cytotoxicity effect on rat cortical neurons were investigated.

The findings indicated that β-CD covalent binding with Cur led to an improvement of its solubility and photophysical properties in aqueous conditions preventing cytotoxic effects. Firstly, UV–vis absorption and fluorescence emission spectra in D_2_O revealed that, compared to free Cur, water solubility of the nanoconjugates increased with the number of β-CD resulting in an improvement of the absorption intensity and in the restoration of fluorescence. Secondly, the addition of β-CD reduced significantly the toxicity of Cur illustrated by around 90% and 80% rat cortical neurons viability for (β-CD)_2_@Cur and β-CD@Cur nanoconjugate, respectively, at 20 µM concentration, compared to only 48% viability with free Cur at the same concentration. These results showed the potential interest of these nanoconjugates as possible new Cur derivatives for application in neurodegenerative diseases prevention. The next step will be to determine the efficacy of curcumin’s neuroprotective effects of these two nanoconjugates, including protection against Aβ cell toxicity, and antioxidative properties using cell and animal models, with the goal towards developing prevention strategies to reduce risk of neurodegenerative diseases. 

## Figures and Tables

**Table 1:** Photophysical properties of Cur and nanoconjugates **4** and **5**.

Compounds	λ_abs_, _max_ (nm)	λ_em, max_ (nm)	ϕ_f_	τ (ns)
Cur	428	~580	<<0.01	1.6
β-CD@Cur **4**	426	580	0.03	2.8
(β-CD)_2_@Cur **5**	365	507	0.09	6.4

## Data Availability

Data is contained within the article or supplementary material.

## Supplementary Materials

The following are available online at https://www.mdpi.com/1422-0067/22/6/3255/s1, Figure S1: ^1^H NMR spectrum (300 MHz, DMSO-*d*_6_, 298 K) of β-CD@Cur **4** nanoconjugate, Figure S2: *J* mod ^13^C NMR spectrum (75 MHz, DMSO-*d*_6_, 298 K) of β-CD@Cur **4** nanoconjugate, Figure S3: 2D HSQC NMR spectrum (300 MHz, DMSO-*d*_6_, 298 K) of β-CD@Cur **4** nanoconjugate, Figure S4: 2D HMBC NMR spectrum (300 MHz, DMSO-*d*_6_, 298 K) of β-CD@Cur **4** nanoconjugate, Figure S5: ^1^H NMR spectrum (300 MHz, DMSO-*d*_6_, 298 K) of (β-CD)_2_@Cur **5** nanoconjugate, Figure S6: *J* mod ^13^C NMR spectrum (75 MHz, DMSO-*d*_6_, 298 K) of (β-CD)_2_@Cur **5** nanoconjugate, Figure S7: 2D HSQC NMR spectrum (300 MHz, DMSO-*d*_6_, 298 K) of (β-CD)_2_@Cur **5** nanoconjugate, Figure S8: 2D HMBC NMR spectrum (300 MHz, DMSO-*d*_6_, 298 K) of (β-CD)_2_@Cur **5** nanoconjugate.

## Abbreviations

Cur	Curcumin
β-CD	β-Cyclodextrin
CuAAC	Copper-catalyzed azide–alkyne cycloaddition
BBB	Blood Brain Barrier
AD	Alzheimer’s disease
