# Supplementary material for: Synthesis of New Water Soluble β-Cyclodextrin@Curcumin Conjugates and In Vitro Safety Evaluation in Primary Cultures of Rat Cortical Neurons
Source: Int J Mol Sci. 2021 Mar 23;22(6):3255. doi: 10.3390/ijms22063255 (PMC8004725; doi:10.3390/ijms22063255)
Supplement: Supplementary file 1 [file ijms-22-03255-s001.pdf]

International Journal of *Molecular Sciences*

MDPI

Supplementary Materials

Synthesis of new water soluble β-Cyclodextrin@Curcumin conjugates and *in vitro* safety evaluation in primary cultures of rat cortical neurons

Amina Ben Mihoub [1,2,3,*], Samir Acherar [2,*], Céline Frochot [3], Catherine Malaplate [4], Frances T. Yen [4] and Elmira Arab-Tehrany [1,*]

[1] LIBio Laboratory, Université de Lorraine, F-54000 Nancy, France
[2] LCPM, CNRS, Université de Lorraine, F-54000 Nancy, France
[3] LRGP, CNRS, Université de Lorraine, F-54000 Nancy, France; celine.frochot@univ-lorraine.fr
[4] URAFPA, INRAE, Université de Lorraine, F-54000 Nancy, France; catherine.malaplate-armand@univ-lorraine.fr (C.M.); frances.yen-potin@univ-lorraine.fr (F.T.Y.)
* Correspondence: amina.ben-mihoub@univ-lorraine.fr (A.B.M.); samir.acherar@univ-lorraine.fr (S.A.); elmira.arab-tehrany@univ-lorraine.fr (E.A.-T.)

Table of contents

Characterization of Cur mono-alkyne **1** — **S-2**

Characterization of Cur di-alkyne **2** — **S-3**

1D and 2D NMR spectra (^{1}H, ^{13}C, HSQC and HMBC) of β-CD@Cur **4** nanoconjugate — **S-4**

1D and 2D NMR spectra (^{1}H, ^{13}C, HSQC and HMBC) of $(\beta\text{-CD})_2$@Cur **5** nanoconjugate — **S-9**

International Journal of
Molecular Sciences

MDPI

Characterization of Cur mono-alkyne 1

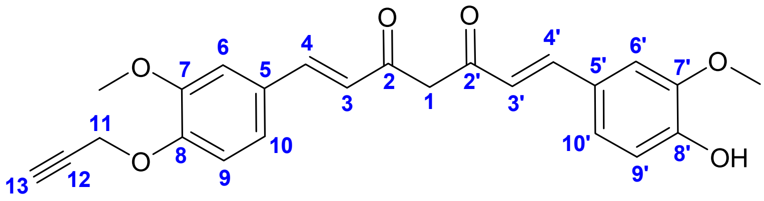

- **TLC**: R_f = 0.63 (EtOAc/Hexane = 50:50, v/v)

- **Analytical HPLC**: Rt = 12.62 min (ACN/H_2O, v/v) gradients from 10/90 to 100/0 in 25 min, then 100/0 for 10 min containing 0.1 % trifluoroacetic acid (TFA). A flow rate of 1.0 mL/min with UV detection at 254 nm and 420 nm.)

- **^{1}H NMR (300 MHz, DMSO-*d*$_6$)**: δ 3.59 (t, *J* = 2.1 Hz, 1H, H_{13}), 3.84 (s, 6H, $H_{7\text{-OMe}}$ and $H_{7'\text{-OMe}}$), 4.85 (d, *J* = 2.1 Hz, 2H, H_{11}), 6.10 (s, 2H, H_1), 6.79 (d, *J* = 1.8 Hz, 1H, H_6), 6.83 (d, *J* = 8.1 Hz, 1H, H_9), 6.87 (d, *J* = 1.8 Hz, 1H, $H_{6'}$), 7.08 (d, *J* = 8.4 Hz, 1H, $H_{9'}$), 7.16 (dd, *J* = 7.8 and 1.8 Hz, 1H, H_{10}), 7.27 (dd, *J* = 8.7 and 1.8 Hz, 1H, $H_{10'}$), 7.35 (d, *J* = 15.6 Hz, 2H, H_3 and $H_{3'}$), 7.57 (d, *J* = 15.6 Hz, 2H, H_4 and $H_{4'}$), 9.65 (s, 1H, $H_{8'\text{-OH}}$)

- **HRMS (ESI) for $C_{24}H_{22}O_6$**: $[M+H]^+$ calculated 407.1494, found 407.1867 and $[M+K]^+$ calculated 445.1053, found 445.1531

The physical data (NMR and HRMS) are in agreement with the values reported in the literature [1].

Characterization of Cur di-alkyne 2

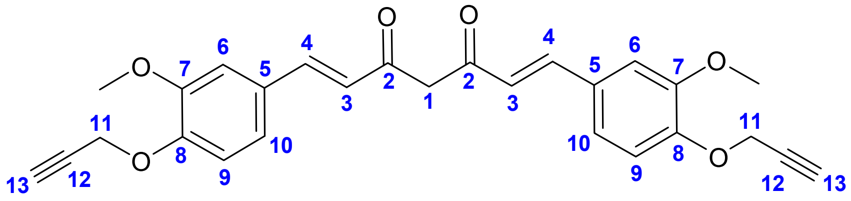

- ✓ **TLC**: R_f = 0.72 (EtOAc/Hexane = 50:50, v/v)
- ✓ **Analytical HPLC**: Rt = 13.64 min (ACN/H_2O, v/v) gradients from 10/90 to 100/0 in 25 min, then 100/0 for 10 min containing 0.1 % trifluoroacetic acid (TFA). A flow rate of 1.0 mL/min with UV detection at 254 nm and 420 nm.)
- ✓ **^{1}H NMR (300 MHz, DMSO-*d*$_6$)**: δ 3.59 (t, *J* = 2.1 Hz, 2H, H_{13}), 3.85 (s, 6H, $H_{7\text{-OMe}}$), 4.86 (d, *J* = 2.1 Hz, 4H, H_{11}), 6.13 (s, 2H, H_1), 6.84 (d, *J* = 1.5 Hz, 2H, H_6), 6.95 (d, *J* = 7.9 Hz, 2H, H_9), 7.19 (dd, *J* = 8.1 and 1.5 Hz, 2H, H_{10}), 7.37 (d, *J* = 16.0 Hz, 2H, H_3), 7.60 (d, *J* = 16.0 Hz, 2H, H_4)
- ✓ **HRMS (ESI) for $C_{27}H_{24}O_6$**: $[M+H]^+$ calculated 445.1651, found 445.1962 and $[M+K]^+$ calculated 483.1209, found 483.1643

The physical data (NMR and HRMS) are in agreement with the values reported in the literature [1]..

International Journal of
Molecular Sciences

MDPI

1D and 2D NMR spectra (^{1}H, ^{13}C, HSQC and HMBC) of β-CD@Cur 4 nanoconjugate

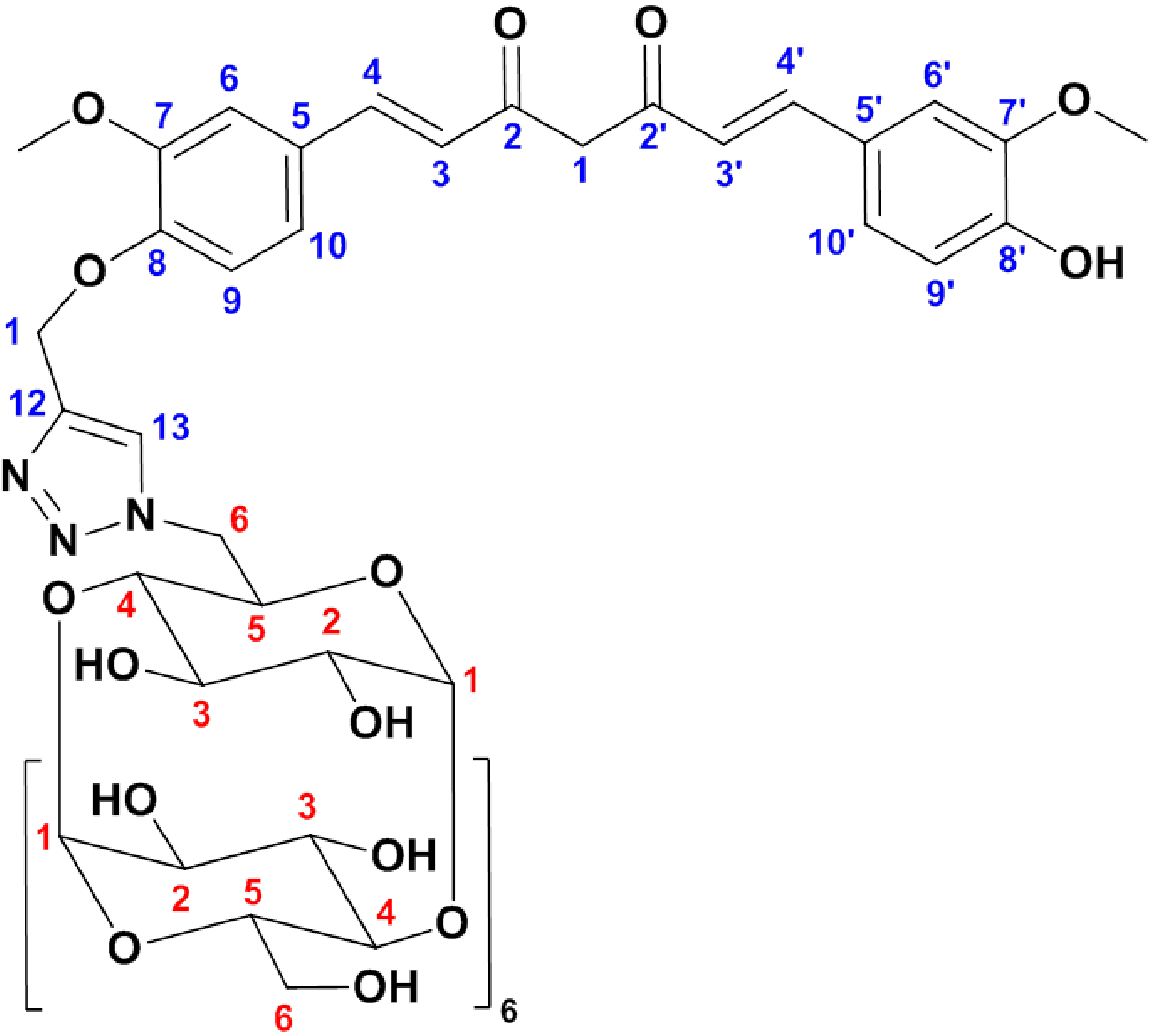

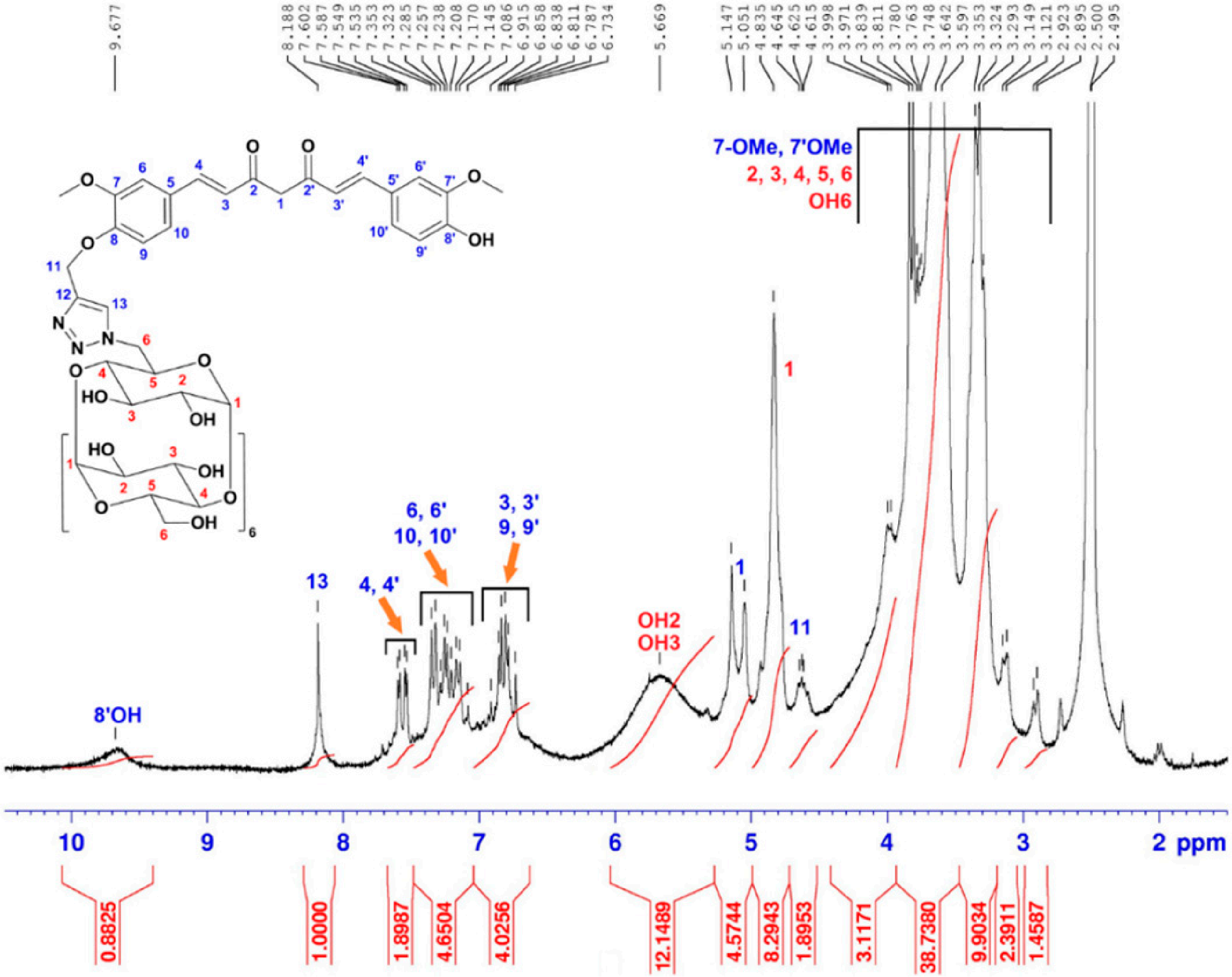

Figure S1. ^{1}H NMR spectrum (300 MHz, DMSO-*d*$_6$, 298K) of β-CD@Cur **4** nanoconjugate.

International Journal of
Molecular Sciences

MDPI

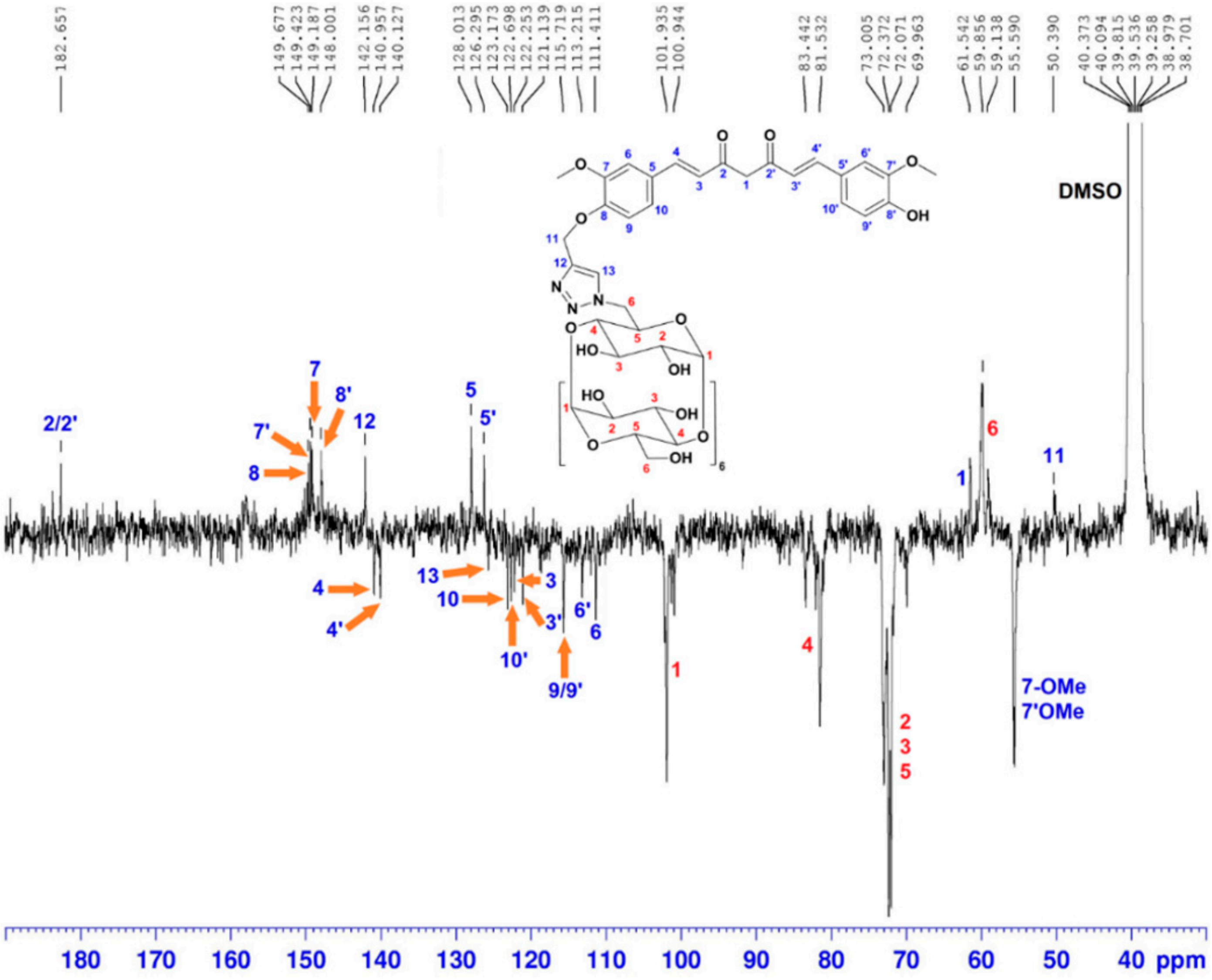

Figure S2. *J* mod ^{13}C NMR spectrum (75 MHz, DMSO-d_6, 298K) of β-CD@Cur **4** nanoconjugate (C and CH_2 up; CH_3 and CH down).

International Journal of
Molecular Sciences

MDPI

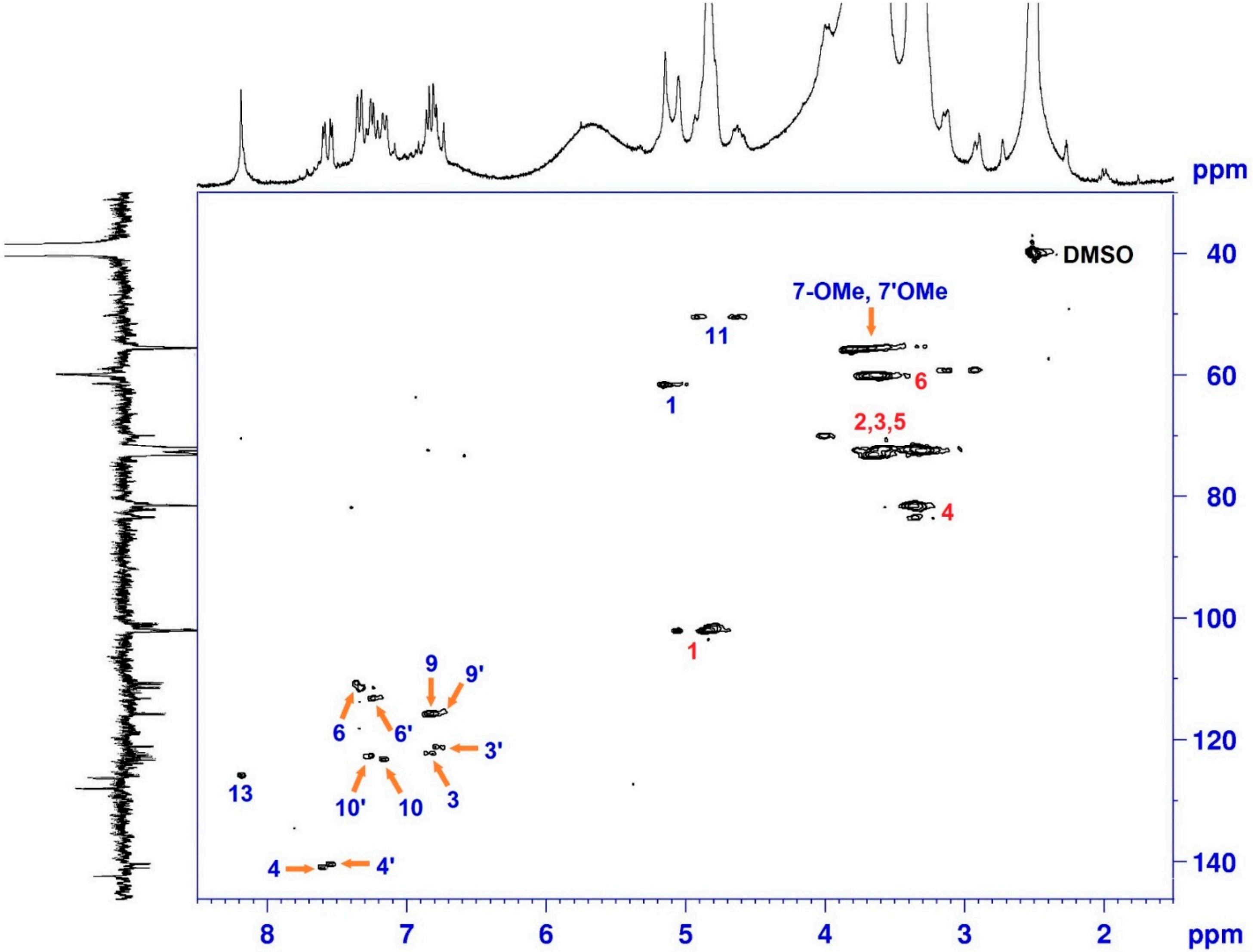

Figure S3. 2D HSQC NMR spectrum (300 MHz, DMSO-d_6, 298K) of β-CD@Cur **4** nanoconjugate.

International Journal of
Molecular Sciences

MDPI

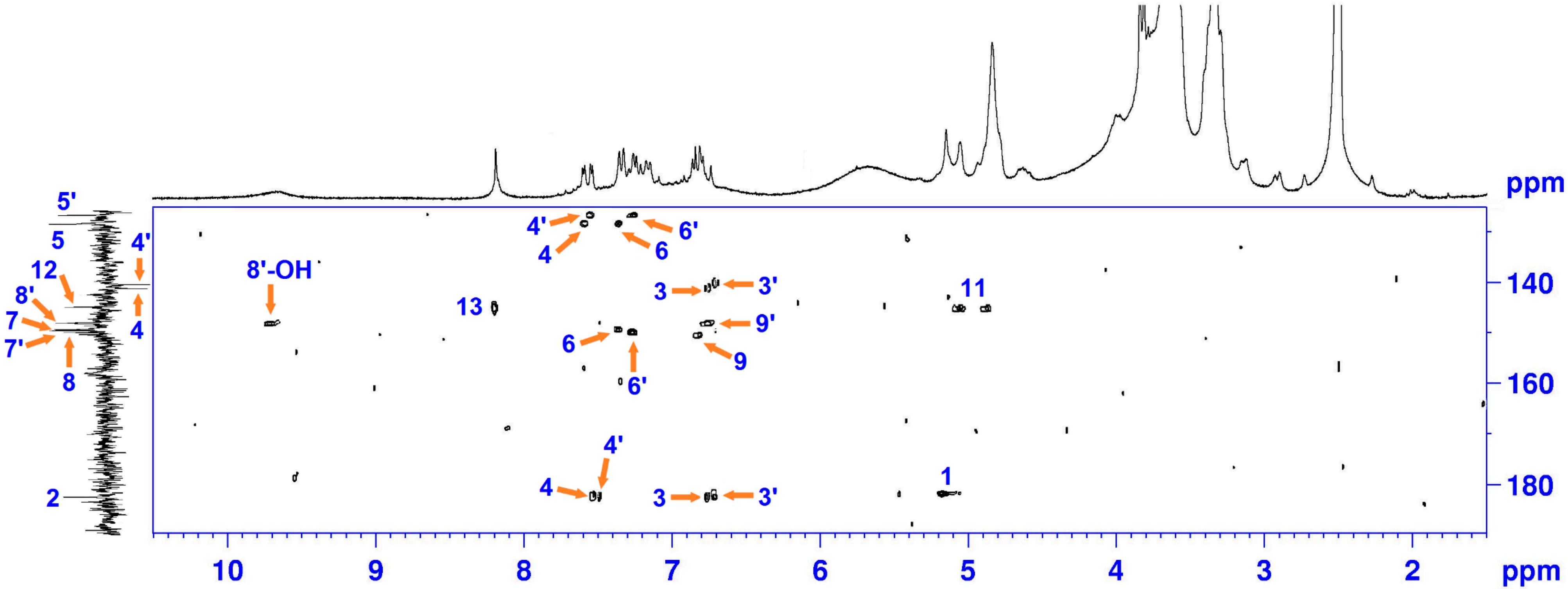

Figure S4. 2D HMBC NMR spectrum (300 MHz, DMSO-*d*$_6$, 298K) of β-CD@Cur **4** nanoconjugate.

International Journal of *Molecular Sciences*

MDPI

1D and 2D NMR spectra (^{1}H, ^{13}C, HSQC and HMBC) of (β-CD)$_2$@Cur 5 nanoconjugate

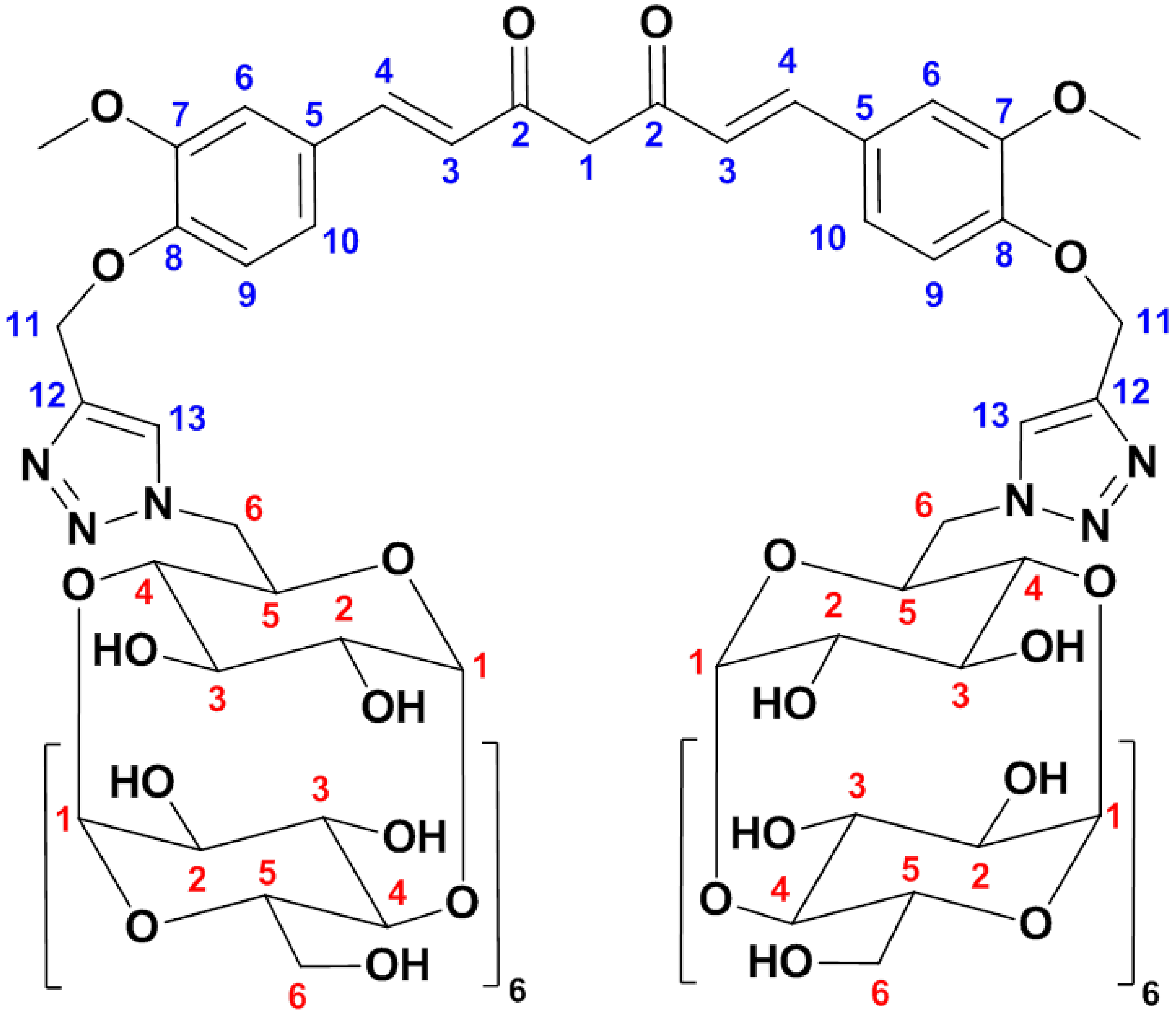

International Journal of
Molecular Sciences

MDPI

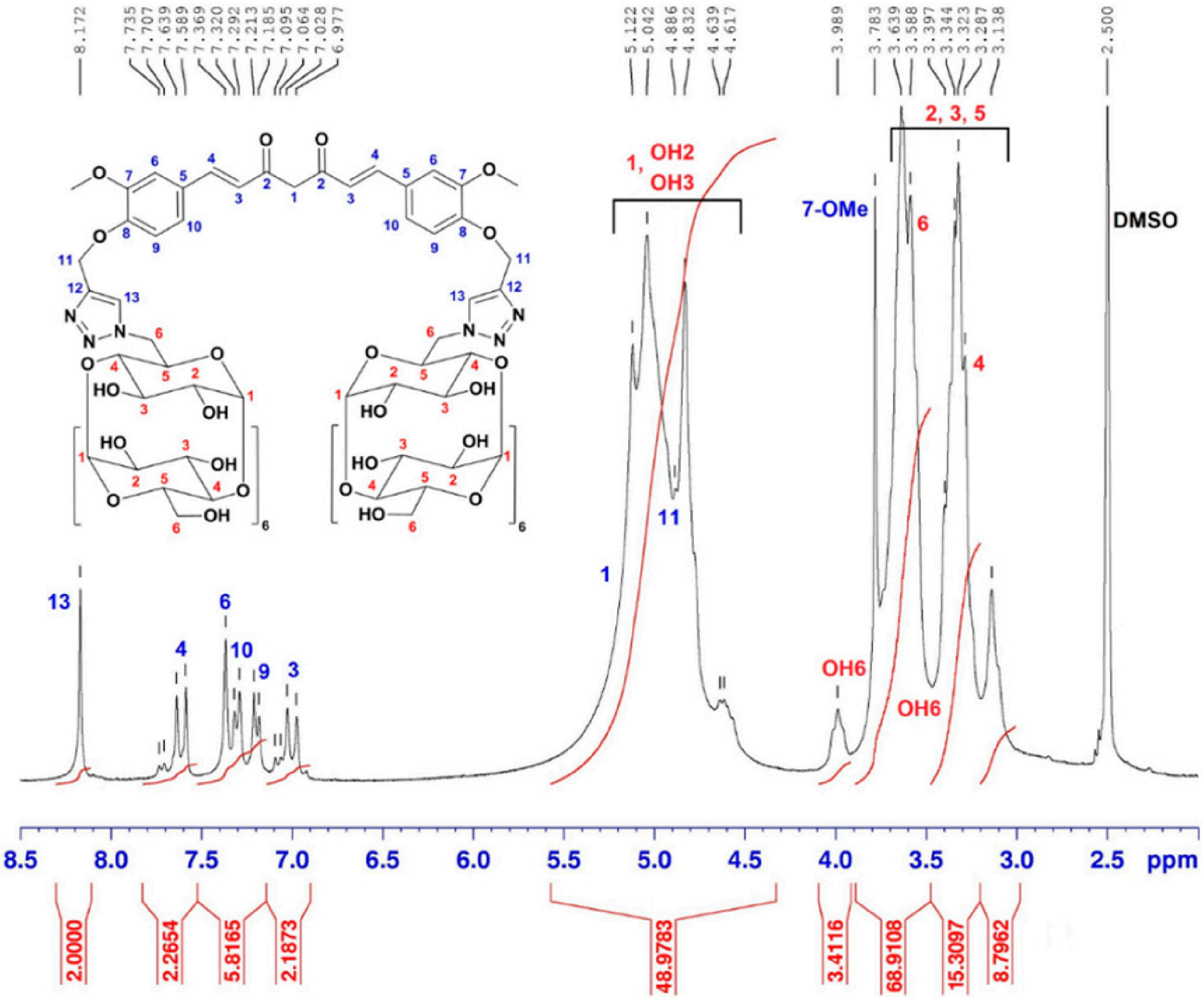

Figure S5. ^{1}H NMR spectrum (300 MHz, DMSO-d_6, 298K) of (β-CD)$_2$@Cur **5** nanoconjugate.

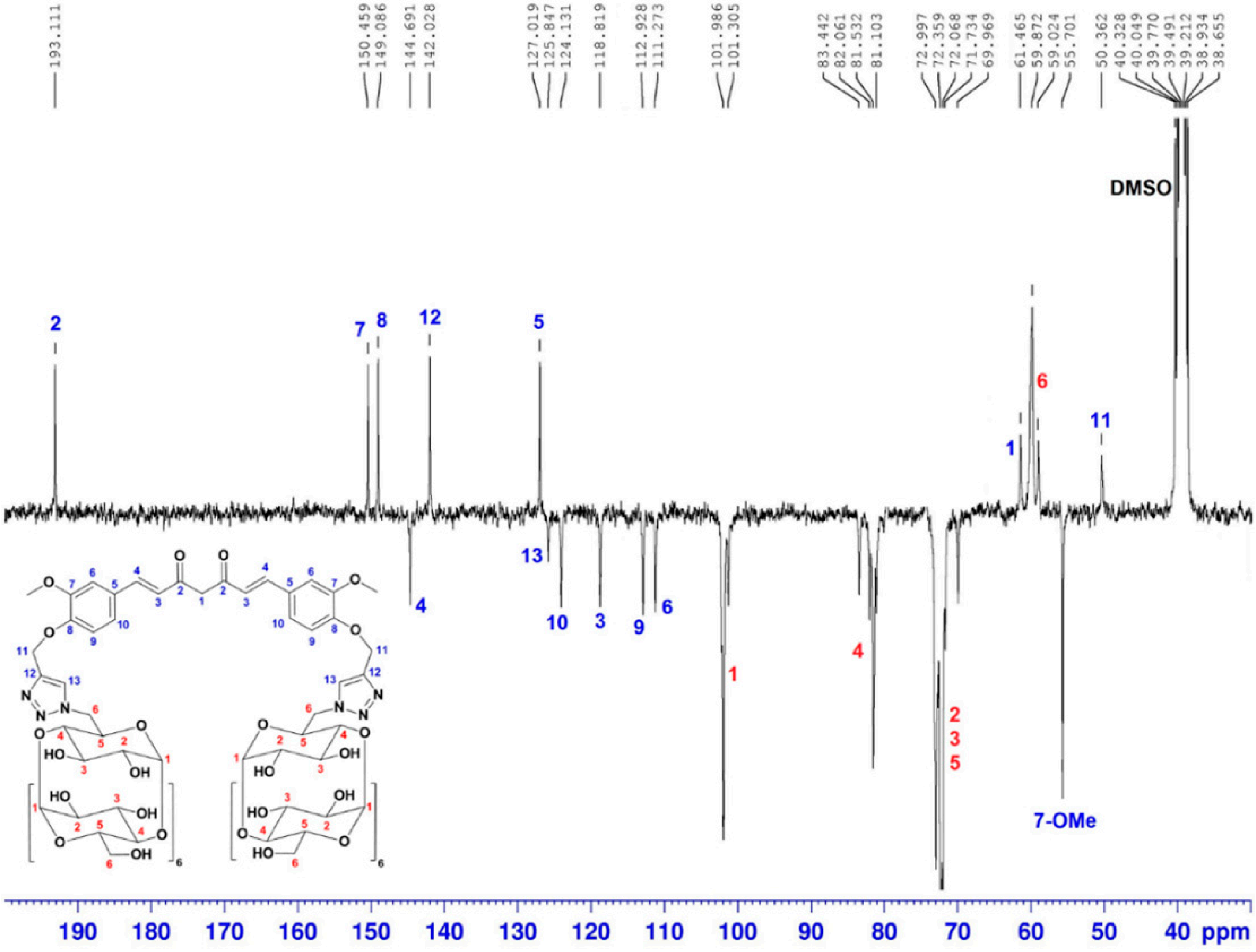

Figure S6. *J* mod ^{13}C NMR spectrum (75 MHz, DMSO-d_6, 298K) of (β-CD)$_2$@Cur **5** nanoconjugate (C and CH_2 up; CH_3 and CH down).

International Journal of
Molecular Sciences

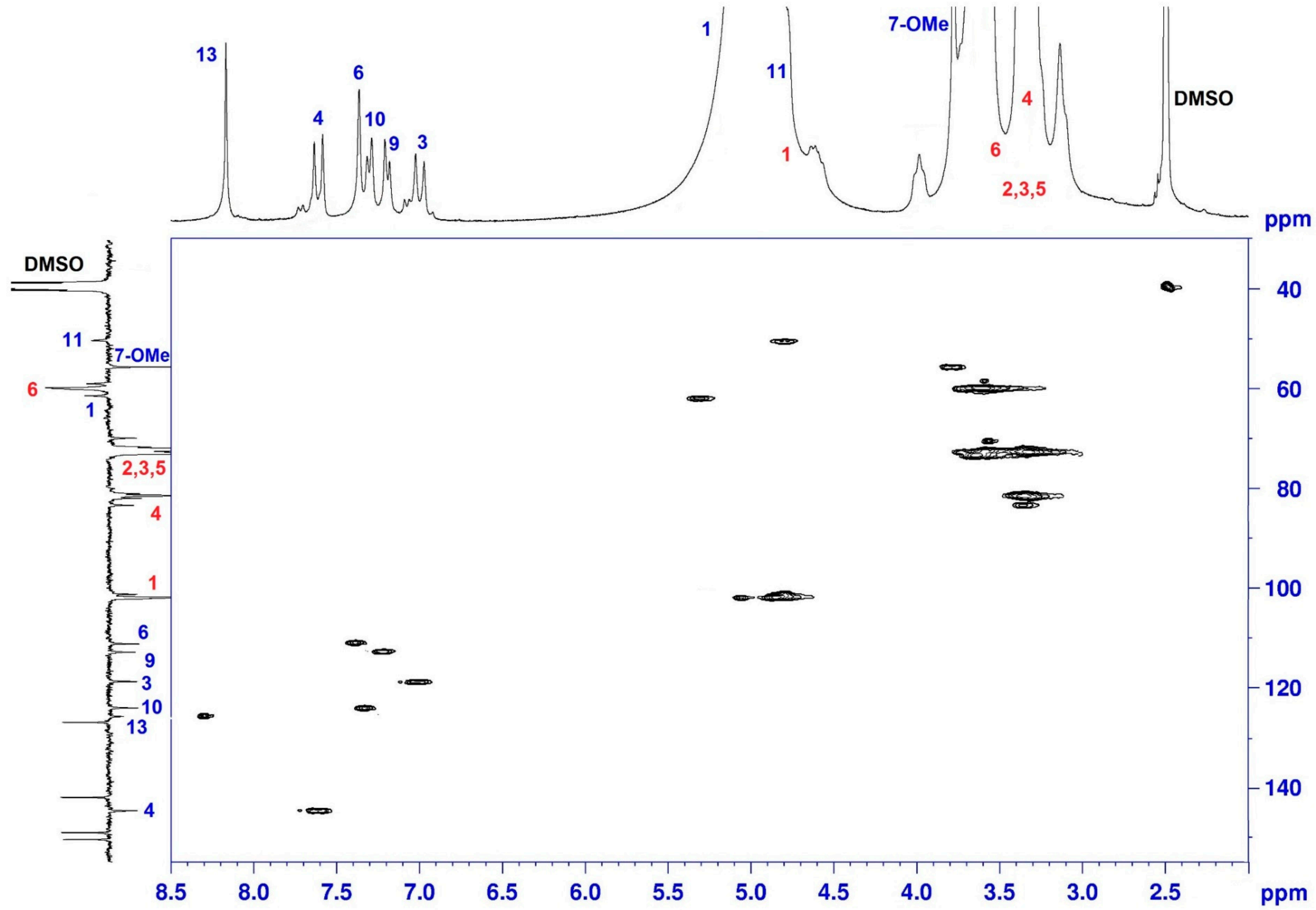

Figure S7. 2D HSQC NMR spectrum (300 MHz, DMSO-d_6, 298K) of (β-CD)$_2$@Cur **5** nanoconjugate.

International Journal of
Molecular Sciences

MDPI

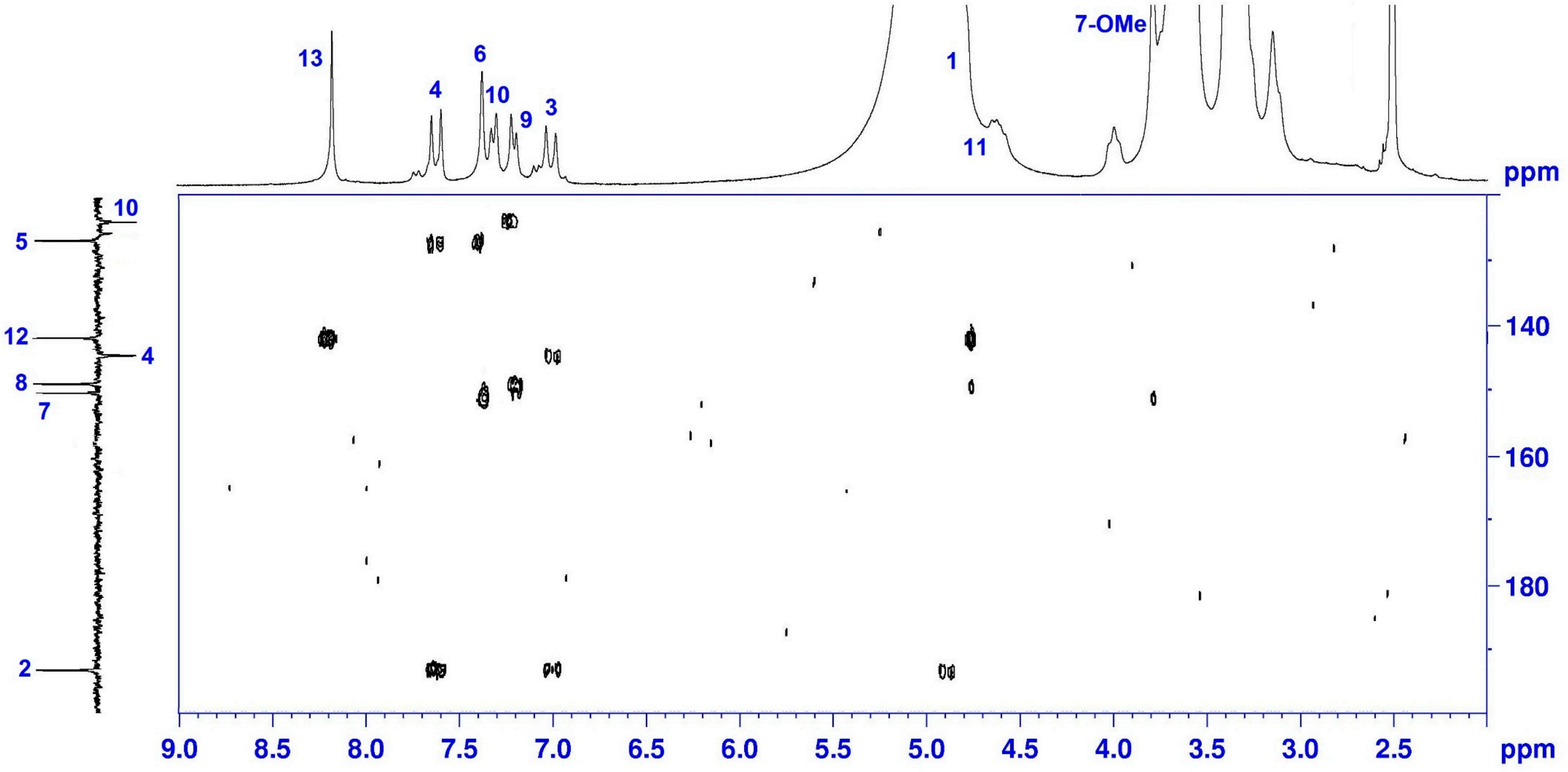

Figure S8. 2D HMBC NMR spectrum (300 MHz, DMSO-d_6, 298K) of (β-CD)$_2$@Cur **5** nanoconjugate.

References

1. Raja, K.; Alonso, A.; Banerjee, P.; Dolai, S.; Corbo, C.; Averick, S.; Mogha, A.; Debnath, S. Curcumin derivatives. WO2011106691A2.**2011**. https://patentimages.storage.googleapis.com/44/6a/79/e7561b802ebbd8/WO2011106691A2.pdf
